# Digital design and evaluation for additive manufacturing of personalized myopic glasses

**DOI:** 10.1038/s41598-022-17233-9

**Published:** 2022-07-28

**Authors:** Jianwen Xu, Bin Liu, Yizhen Wang, Kaiyong Jiang

**Affiliations:** Fujian Provincial Key Laboratory of Special Energy Manufacturing, Xiamen Key Laboratory of Digital Vision Measurement, Huaqiao University, Xiamen, 361021 China

**Keywords:** Engineering, Mathematics and computing

## Abstract

Myopic glasses design has critical effects on the match between glasses and individual face. Improper myopic glasses design may affect the try-on comfort and health. It is difficult for the myopic glasses to be adjusted variedly and suitably from people to people with the limitations of traditional manufacturing processes and current design methods. In this paper, an evaluation descriptor named glasses fit score, which contains alignment scores and design scores, is proposed to guide and evaluate the myopic glasses design. Based on the descriptor, a novel approach is presented to complete the myopic glasses design and manufacturing individually. The approach can be divided into three steps: glasses alignment, glasses personalized design and glasses manufacturing. During the glasses alignment, the myopic glasses are aligned to the face to obtain the alignment score of the descriptor based on the face symmetry plane and feature points, including the silent point of the eye and the top point of the ear. After the glasses alignment, the myopic glasses can be deformed to match the face to achieve the design score of the descriptor. The deformations include glasses frame transformation, glasses leg option and glasses personalized mark. In the glasses manufacturing, the designed myopic glasses can be fabricated by a 3D printer. Then, a post processing process is conducted to polish the myopic glasses with oil painted. Finally, the proposed approach is applied to adjust the myopic glasses for several people. The results show that, compared to the previous methods, the approach can make the myopic glasses aligned and deformed to the individual face effectively to obtain an ideal score of the descriptor, thus improving the match between the glasses and the individual face.

## Introduction

Glasses, a serviceable accessory, are usually used in daily life^1,2^. The match between glasses and individual faces (MGIF) contains sizes and shapes of the glasses with matching the face, e.g., the match of the width between glasses and face, the match of the glasses nose pad and nose. If MGIF is not perfect, for instance, the small width of the glasses, it can make the glasses chuck the face which can affect the try-on comfort. For the myopic glasses, as it has a special function to correct the vision, it should concern both try-on health and comfort. Improper designed myopic glasses may result in try-on comfort problems, for instance, falling from the nose, which can change its position relationship with the face, and affect the corrective vision effect with leading to the try-on health problems.

At present, the methods of glasses design can be divided into two aspects: forward design and reversal design. To the forward design^3–5^, experience with the designer and static data of the face are adopted to design a series of glasses with different styles and specifications. Obviously, the designed glasses cannot consider the individual face data that MGIF may be imperfect for the myopic glasses.

The reversal design^6–11^ methods are carried out to obtain and use 2D or 3D individual face data for designing the glasses. However, most of the current methods are based on virtual try-ons, and the glasses cannot be designed to match the individual faces. Although there are some methods using 3D face data, the evaluation of MGIF and glasses personalized design are not perfect.

In order to solve the try-on problems of current methods, we present an approach to design personalized myopic glasses. The approach is to establish the position relationship between the glasses and the individual face, and then the glasses are adjusted to match the face.

The difficulty of the approach is how to establish an accurate position relationship between the myopic glasses and the face. Considering the myopic glasses has a symmetric feature and the face has an approximate global symmetric feature and local asymmetric features, the global symmetric feature, namely, the symmetry plane based on the local area of the nose with sufficient symmetry features, can be used to establish the position relationship named glasses alignment method, and the local asymmetric features, *e.g.*, the pupil distance, can be used to adjust the myopic glasses.

The challenge of the myopic glasses personalized design is that there is no perfect standard to guide and evaluate it, especially the subjective indicators: try-on health and comfort. Thus, we present an evaluation descriptor named glasses fit score (GFS) to express the subjective indicators. But it is difficult for the subjective indicators to be embodied on the myopic glasses design. In order to build their relationships, the subjective indicators are decomposed into seven key objective indicators, e.g., the distance between glasses and eyes, the match of the geometric central distance of the glasses and the pupil distance of the face, the match of the glasses nose pad and the nose, and quantified which contain alignment and design scores.

The contributions of this paper are summarized as follows:

1. Quantification and evaluation indicators of the myopic glasses design are established, wherein, the subjective indicators describing try-on health and comfort of the myopic glasses are composed into seven key objective indicators by a descriptor glasses fit score (GFS).
2. A novel digital design and evaluation for additive manufacturing of personalized myopic glasses approach based on 3D individual face data is presented to adjust the myopic glasses to match the face. The global symmetric feature of the face is used to aligned the myopic glasses, and the local asymmetric features of the face are used to adjust the myopic glasses.
3. A novel glasses alignment method is proposed by using the face symmetry plane based on the local area of the nose. As it’s no necessary to use a complete face data, the alignment efficiency can be greatly improved.

## Related work

Current research on the glasses design contains forward design and reversal design.

In the first aspect of the glasses design, Niu et al.^3^ carried out a three dimensional style design of multifunction glasses based on Pro/E software. Based on the design relevance between the general design and the myopic glasses, Ding and Wei^12^ designed variable myopic glasses that were suitable for different shape parameters of faces to make the furthest need of users’ individuality and variety. Ma et al.^4^ used a top-to-down design technology to design the glasses by analysing the structure of the glasses and using UG software. Liu et al.^13^ built a parametrization design mechanism of the glasses based on the measure size and model drive of 3D faces under the condition of measurement data of Chinese face data. Xu^14^ developed a design system of glasses by utilizing the feature match of the glasses style cognize and the genetic algorithm of the glasses layer design way. Xu^5^ built a design wizard system of the glasses based on the knowledge drive of UG software to decrease the dependence of the designer. Thus, the design efficiency was improved.

According to the forward design, different styles and specifications of the glasses can be designed based on experience. To the glasses without the need of vision correction, *e.g.*, the sun glasses, the designed glasses can meet the need of people. However, it has limitations for the myopic glasses. Obviously, the designed glasses have symmetric features, *e.g.,* the geometric central distance. There can be inconformity between the glasses and the individual faces, because the feature parameters of individual faces are significantly different, *e.g.*, the differences of the pupil distance, which can make the distinction of the feature parameters between the individual faces and the glasses. Hence, MGIF can be imperfect and affect the comfort and health of try-ons for the myopic glasses.

The reversal design can be summarized with 2D based and 3D based.

For the 2D based aspect, Jiang^15^ designed an intelligent glasses equipment to realize the face detection function and show the result of the glasses try-on. Tan^6^ gained 2D face data based on computer vision technology and designed a glasses virtual try-on system by combining face detection and 3D model processing technology. Young et al.^7^ predicted the best style of glasses and showed virtual try-on results based on face images and depth convolutional neural networks. Milanova and Aldaeif^16^ presented a virtual eye glasses try-on system that detected and tracked human faces and eyes for people to select 3D virtual glasses. But it is limited to the glasses virtual try-on with 2D images. The myopic glasses cannot be designed to achieve a good MGIF.

For the 3D based aspect, Zhou^17^ adopted a 3D scanner to obtain a point cloud of the glasses. Then, reversal engineering software was applied to redesigning the glasses. Because it doesn’t consider the individual face data, MGIF cannot be perfect for the myopic glasses. Patil et al.^8^ designed a glasses virtual try-on system to conduct detection of individual faces and eyes for estimating a 3D face model. Martin and Batista^9^ presented an automation detection frame of face attitude based on a single image to gain a 3D rigid model of the face. Yamamoto et al.^18^ proposed a try-on system that can help users choose glasses frames and lenses by rendering users’ 3D faces. However, the 3D face is not used to design the myopic glasses. Liu^10^ used a 3D scanner to obtain the head information for building a digital head model. Parameterization can be provided for users to revise the selected glasses and analogue simulation between the glasses and head model. However, the glasses are designed based on some face features, and the feature data of the face cannot be excavated deeply to design the glasses. Thus, MGIF is imperfect for the myopic glasses. Wang^11^ built a mathematical relation between face features and key design feature parameters of glasses to carry out parametrization design. Although some feature parameters of 3D face can be obtained to design the glasses, the parameters are insufficient, especially the pupil distance, which can lead to an imperfect MGIF for the myopic glasses.

It can be concluded that although current methods can meet the basic needs of the glasses design, they have limitations. For the forward design, as the glasses are designed based on experience and cannot consider the individual face data, MGIF may not be good, which may result in poor comfort of try-ons for the myopic glasses. For the reversal design, it is easy to obtain 3D face data with the development of 3D scanning technology and equipment. However, 3D face data are incomplete, and most current methods focus on virtual try-ons. Although there are some methods based on the features of 3D face data, the research between match evaluation and individualized design is not sufficient and deep, and cannot achieve a perfect MGIF for the myopic glasses.

To obtain a perfect MGIF, a digital design and evaluation for additive manufacturing of personalized myopic glasses approach is proposed with glasses virtual assembly and personalized design based on symmetry plane and feature shape and size of the face, which can improve MGIF to enhance the health and comfort of the myopic glasses try-on.

## Methods

**Figure 1:**
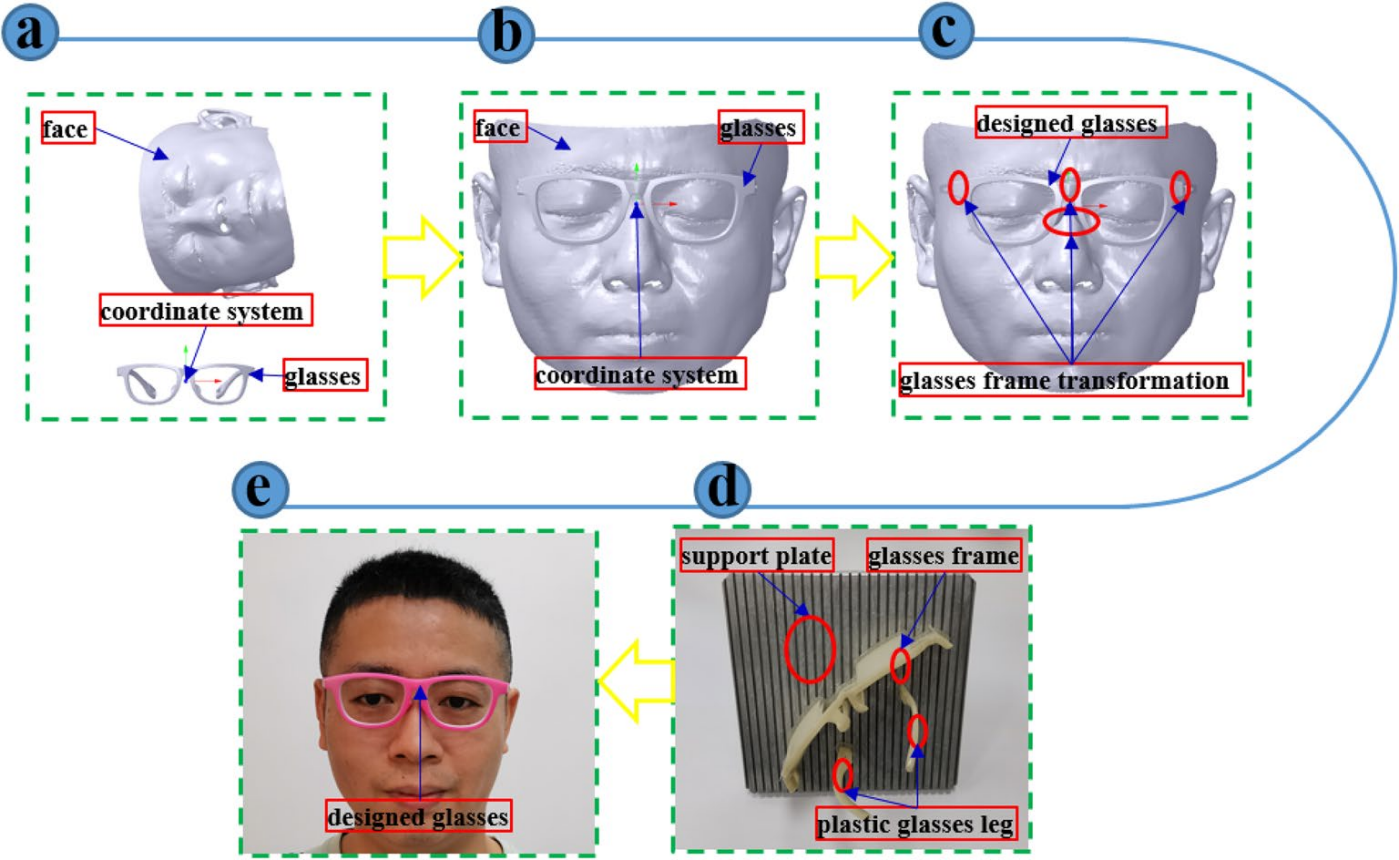
Pipeline of digital design and evaluation for additive manufacturing of personalized myopic glasses (a) Original assembly (b) Glasses alignment (c) Glasses personalized design (d) Glasses manufacturing (e) Try-on result of the myopic glasses.

The approach pipeline can be shown in Fig. 1. It can be mainly divided into three steps: glasses alignment, glasses personalized design and glasses manufacturing.

### Glasses alignment (Fig. 1b)

Glasses alignment can be summarized with three steps: symmetry alignment, coordinate system alignment and revised alignment. During the symmetry alignment, the reflective symmetry plane of the face is extracted to align to *YOZ* plane. In the coordinate system alignment, *X* axis alignment is conducted to align the silent point of the eye to the geometric central line at the position of the pupil height line of the glasses frame. Then, *XOZ* plane alignment is carried out to align the face according to a referring plane passing through the silent point of the eye and vertical to *YOZ* plane. Subsequently, the face is translated to keep a suitable distance between glasses and eyes to obtain the alignment score of GFS. During the revised alignment, height consistency between the inflection point of the plastic glasses leg and the top point of the ear is applied to revise the coordinate system alignment.

### Glasses personalized design (Fig. 1c)

Glasses personalized design containing glasses frame transformation, plastic glasses leg option and glasses personalized mark is carried out to obtain the design score of GFS. During the glasses frame transformation, glasses frame is translated based on the geometric central distance of the glasses and the pupil distance of the face, and rotated to match the dip angle of the face. Then, the nose pad is transformed to the nose with translation and rotation. Finally, pile head rotation is conducted to ensure the consistency between the widths of the glasses frame and the face. In the plastic glasses leg option, the glasses leg is selected to maintain the consistency between the length of the glasses and the face. In the glasses personalized mark, a series of features are respectively transferred on the frame and plastic glasses legs.

### Glasses manufacturing (Fig. 1d)

The designed glasses are fabricated by the 3D printer and polished by the grinder and painted.

### Method declarations

All methods were carried out in accordance with relevant guidelines and regulations.

### Informed consent statement

Informed consent was obtained from all participants for participating in the study.

### Ethics declarations

The Huaqiao University Ethics Committee reviewed and approved the study protocol.

## Definition of the glasses fit score

It is necessary to define the components and coordinate system of the myopic glasses to successfully represent GFS and conveniently conduct the myopic glasses design.

### Glasses component

**Figure 2:**
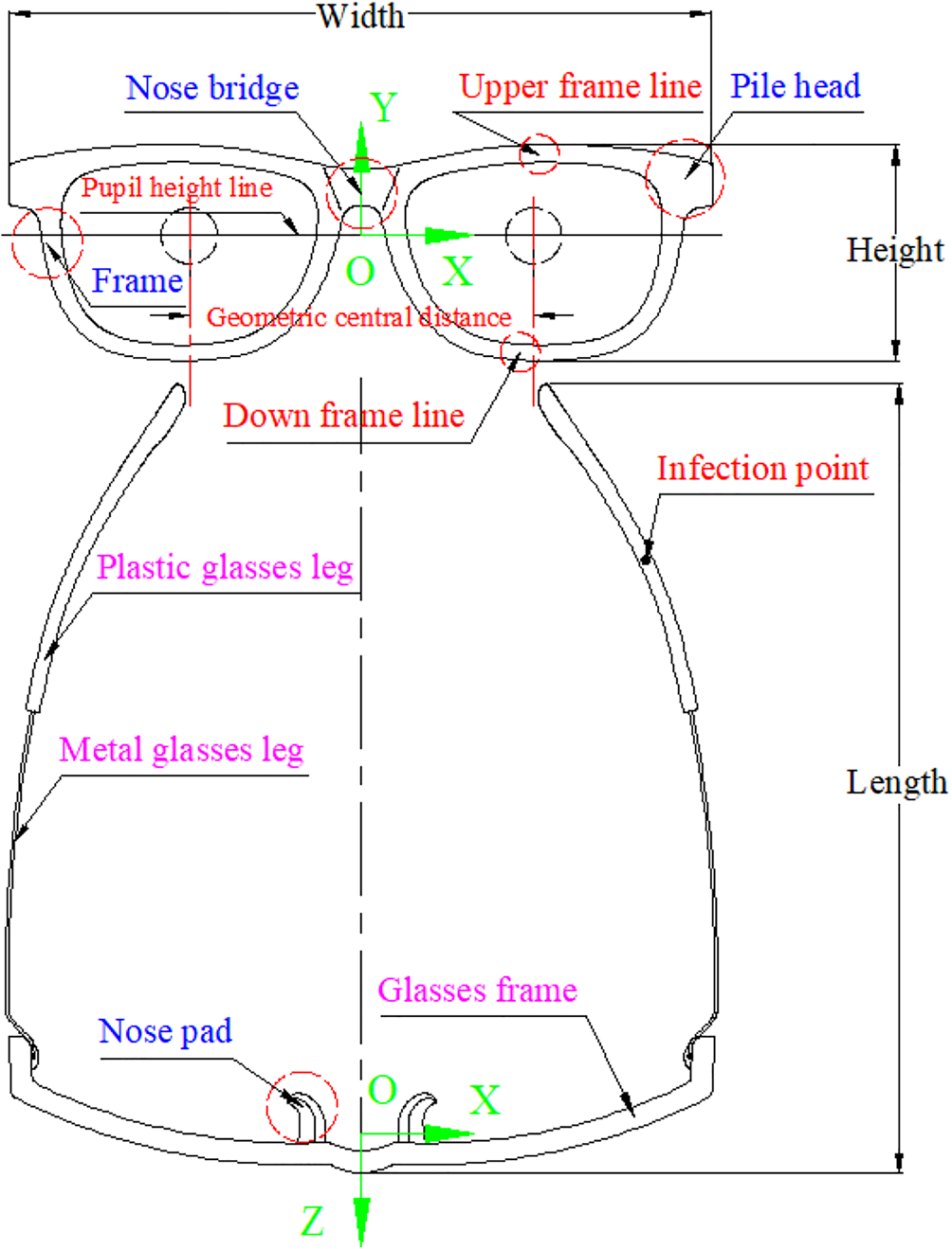
The components and coordinate system of the myopic glasses.

The components of the myopic glasses contain glasses frame, metal glasses legs and plastic glasses legs, as shown in Fig. 2 with pink words. The glasses frame contains frame, nose bridge, nose pad and pile head, as shown in Fig. 2 with blue words. The geometric central distance at the position of the pupil height line of the glasses frame is the base of the glasses alignment, as shown in Fig. 2 with red words.

**Figure 3:**
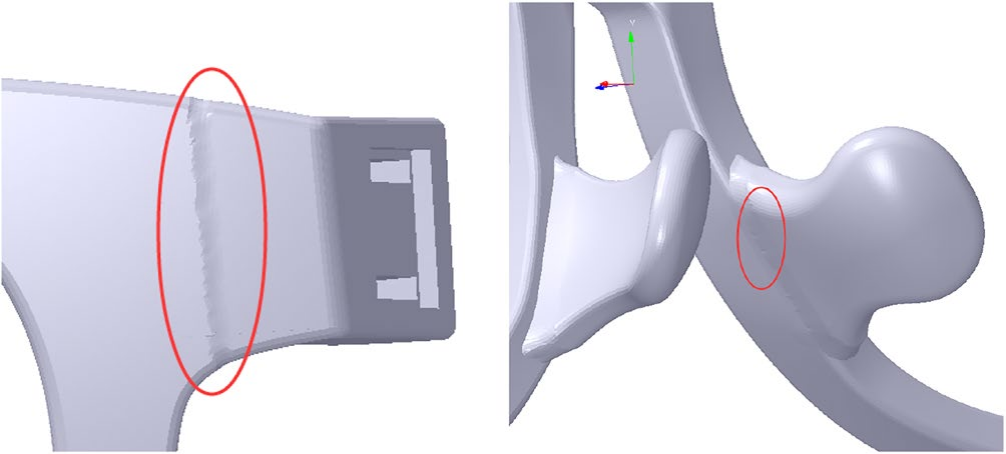
The connection relationship problems at the deformation position marked with red ellipse. From left to right: deformation at the pile head and deformation at the nose pad.

When the glasses frame is deformed as a whole, it may go wrong with several connection relationship problems at the deformation position. On the one hand, when the dip angle of the glasses is adjusted, there may be a serious distortion at the connection relationship between the frame and the pile head, as shown in the left map of Fig. 3. On the other hand, when the nose pad is deformed with a great deformation, smoothing at the deformation area may also be imperfect, as shown in the right map of Fig. 3.

**Figure 4:**
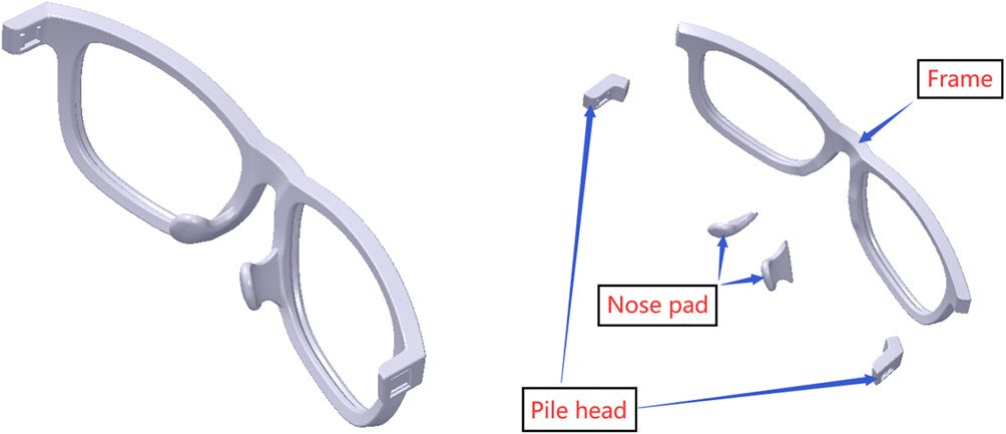
Glasses frame modularization. From left to right: assembly view, disassembly view.

Therefore, a modularization method is applied in the process of the glasses personalized design to address these problems. The glasses frame is selectively divided into three parts, including the frame, the nose pad and the pile head, which are shown in Fig. 4. Each part of the glasses frame is deformed first. Then, the Boolean operation can be carried out to make them as a whole.

### Glasses coordinate system

The coordinate system of the glasses is defined to be located in the middle of the geometric central line at the position of the pupil height line of the myopic glasses, which can make the glasses alignment carried out conveniently and rapidly, as shown in Fig. 2, wherein the cross lines are the geometric centers. *X*, *Y* and *Z* axes of the myopic glasses are along the direction of the geometric central line of the glasses frame, height and length of the glasses, respectively.

### Glasses fit score

**Figure 5:**
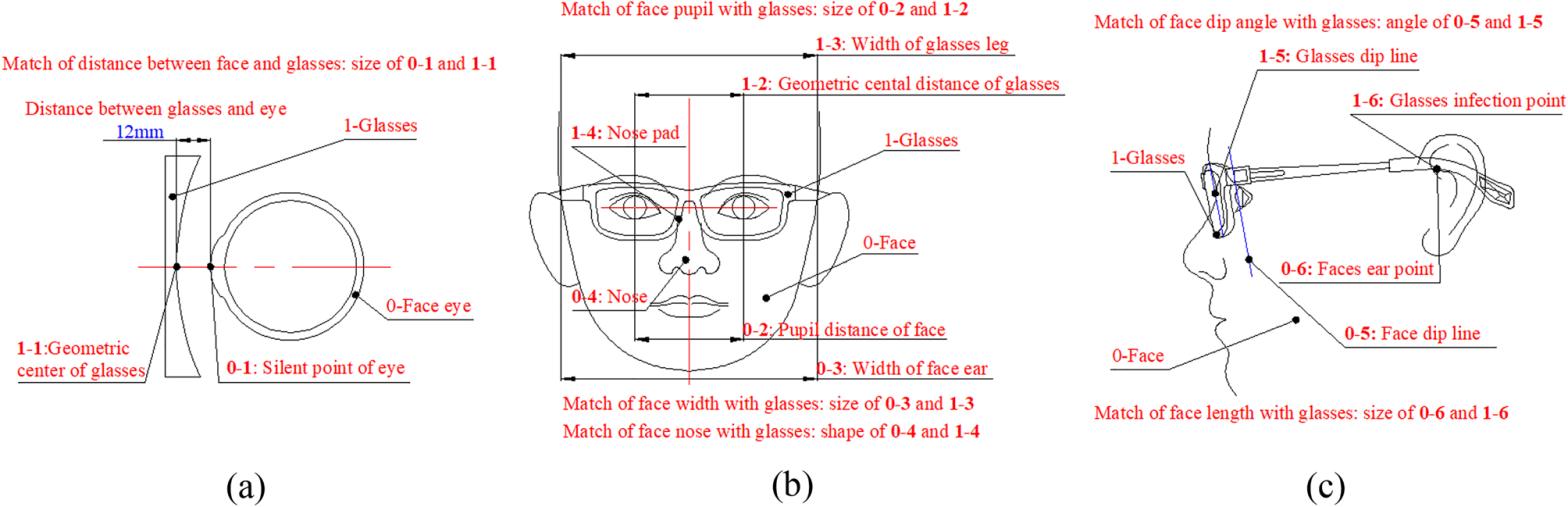
The match between the face and the myopic glasses. (**a**) The match of distance between glasses and eye. (**b**) The match of face pupil, width and nose with glasses. (**c**) The match of face dip angle and length with glasses.

The ideal GFS should make the need for the conditions that are first try-on of health and then comfort^19,20^ for the myopic glasses. For the first condition, on the one hand, the distance between the glasses and the eye should be kept at a proper distance, that is, 12 mm, as shown in Fig. 5a. On the other hand, the geometric central line at the position of the pupil height line of the glasses should be aligned to the pupil distance line of the face, and the geometric central distance should be the same as the pupil distance, as shown in Fig. 5b. The comfort condition includes matching the width, the nose pad, the dip angle and the length with the face, which can be shown in Fig. 5b and Fig. 5c. Each item can be described with a certain score according to its importance for quantifying GFS containing alignment scores and design scores, whose full score is 100, as shown in Table 1.

**Table 1:** GFS quantification.

Item	Step	Match type	Ideal value	Score
M-DGE	Alignment	Size	11 mm	25
M-GP	Design	Size	–	25
M-NN	Design	Shape	–	15
M-PF	Design	Size	3–6°	10
M-DGF	Design	Size	0	10
M-LGF	Design	Size	0–5	10
AG	Design	–	–	5
	Total			100

#### Match of distance between glasses and eye (M-DGE)

It is a size match type, which can be presented the distance between the silent point of the eye and the geometric center of the glasses should be kept with a proper value, that is, 12 mm^20–22^. As the laser light of the scanner can stimulate the eye, the face is scanned with closed eyes, and the value is subtracted by the thickness of the eyelid approximately 1 mm. Hence, the ideal value is 11 mm. Long or short distances can affect the correction vision, leading to the eye health. Its score can be defined as 25, which can be subtracted from 1 by every deviation of 0.25 mm.

#### Match of geometric central distance and pupil distance of face(M-GP)

It is a size match type, which can be represented the geometric central distance should be the same as the pupil distance, and the geometric central line at the position of the pupil height line of the glasses should be aligned to pupil distance line of the face^23–25^. A great error can cause visual fatigue, deepening the myopic degree. Its score can be defined as 25, which can be subtracted from 1 by every deviation of 0.5 mm.

#### Match of nose pad and nose (M-NN)

It is a shape match type, which can be defined the nose pad should have a well match with the nose^12,26^. The match affects the support of the glasses. A bad match can cause the fall of the glasses, leading to an imperfect try-on comfort. Its score can be defined with 15. The score, subtracted from a bad match between the nose pad and the nose, can be defined from two aspects, including the try-on statement and the comfort, as shown in Table 2. Try-on statement is whether the nose pad slides from the nose or not.

**Table 2:** MNPN quantification.

Grade	Sliding from nose	Comfort	Score
1	No	Perfect	15
2	No	Slightly perfect	12
3	No	Imperfect	9
4	Yes	Perfect	6
5	Yes	Imperfect	3

#### Match of pile head and face (M-PF)

It is a size match type, which can be expressed the distance between the plastic glasses legs, which can be shown with the angle between the plastic glasses leg and the ear^12,27^. The ideal angle is 3–6°. It can affect the clamping force between the glasses and the face. A small angle may result in a low clamping force, which can cause the glasses to fall. A large angle may result in a high clamping force, leading to a bad try-on comfort. Its score can be defined as 10, which can be subtracted from 1 by every deviation of 1°.

#### Match of dip angle between glasses and face (M-DGF)

It is a size match type, which can be listed the dip angle of the glasses should match that of the face^12^. A large angle can affect try-on comfort, that is, the contact between glasses and individual faces with a great expression. Its score can be defined as 10, which can be subtracted from 1 by every deviation of 2°.

#### Match of length between glasses and face (M-LGF)

It is a size match type, which can be shown the length of glasses should have a well match with the face, that is, the match between the plastic glasses leg and the ear^21,28^. The ideal length of the plastic leg is 0–5 mm. A long leg may affect try-on comfort with slack statements. A short leg may affect try-on comfort with tight statements. Its score can be defined as 10, which can be subtracted from 1 by every deviation of 1 mm.

#### The appearance of glasses (AG)

It can affect try-on effects^28,29^. Its score can be defined with 5.

## Glasses alignment

**Figure 6:**
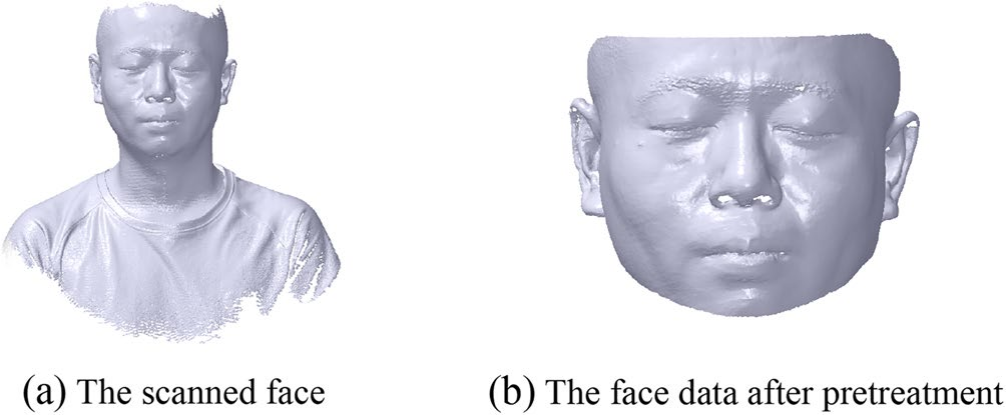
Pretreatment of scanned face.

The 3D face data is obtained by a 3D scanner, as shown in Fig. 6a. While scanning, the scanner software has a function to remove the noise points with outlier from the face. But it is difficult to scan the small features, *e.g.*, the hair, so there are some missing parts in the area of these features. Then the useless data of the face can be trimmed manually, as shown in Fig. 6b. The trimmed standard is to maintain main features of the face, such as the nose, the eye, the ear, and so on.

Before carrying out the myopic glasses design, it is important to select a proper myopic glasses based on the individual face data to first match the width and then the pupil distance of the face^12,30^. Too small or large a size can affect the try-on comfort and health^30^. The width and pupil distance of the face are approximately 135 mm and 66 mm, respectively, with the myopic degree 400 of the left and right eyes; thus, the glasses with a width of 137 mm and a geometric central distance of 67.1 mm are selected.

### Symmetry alignment

**Figure 7:**
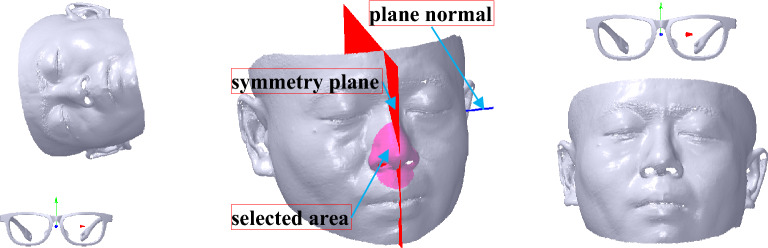
Face symmetry alignment. From left to right: original assembly, symmetry extraction with red plane and blue normal, symmetry alignment result.

The initial coordinate system of the face is obviously inconsistent with the myopic glasses, as shown in the left map of Fig. 7. A method proposed by Xu et al.^31^ is applied to extract the face reflective symmetry plane (FRSP), as shown with a red plane in the middle map of Fig. 7 and pink selected mesh in the area of nose and blue FRSP normal. As it’s no need to use the complete face data, the efficiency can be improved. Then its normal can be aligned to *X* axis, which is denoted as *N*_*x*_ (1,0,0). The FRSP coefficient can be expressed as *P*_*s*_[*p*_*s*0_ *p*_*s*1_ *p*_*s*2_ *p*_*s*3_] with normal *N*_*s*_(*p*_*s*0_, *p*_*s*1_, *p*_*s*2_). Subsequently, FRSP can be translated to the coordinate system that is represented as *O*(0,0,0), and its normal is rotated to parallel to *X* axis.

#### Translation

During the translation, it is necessary to compute the translation transformation matrix *f*_*s*0_ based on the translation vector *T*_*s*_(*t*_*s*0_, *t*_*s*1_, *t*_*s*2_), which can be defined with *O* projected on FRSP.

For a vertex and plane that are, respectively expressed as *V*_0_(*x*_0_, *y*_0_, *z*_0_) and *P*_0_[*A B C D*], the vertex *V*_*p*_(*x*_*p*_, *y*_*p*_, *z*_*p*_) that is *V*_0_ projected on *P*_0_ can be computed as follows:

1 $$x_{p} = \frac{{(B^{2} + C^{2} )x_{0} - A(By_{0} + Cz_{0} + D)}}{{A^{2} + B^{2} + C^{2} }}$$

2 $$y_{p} = \frac{{(A^{2} + C^{2} )y_{0} - B(Ax_{0} + Cz_{0} + D)}}{{A^{2} + B^{2} + C^{2} }}$$

3 $$z_{p} = \frac{{(A^{2} + B^{2} )z_{0} - C(Ax_{0} + By_{0} + D)}}{{A^{2} + B^{2} + C^{2} }}$$

Therefore, the vertex *V*_1_(*x*_1_, *y*_1_, *z*_1_) with *O* projected on FRSP can be computed according to Eqs. (1), (2) and (3) based on *P*_*s*_. Thus, *T*_*s*_ can be obtained with *T*_*s*_ (− *x*_1_, − *y*_1_, − *z*_1_).

Subsequently, the face mesh can be translated according to *f*_*s*0_. For a vertex *V*(*x*, *y*, *z*) transformed with a transformation matrix *f*, its coordinate value *V*′(*x*^′^, *y*^′^,*z*′) can be computed as follows:

4 $$\left[ {\begin{array}{*{20}c} {x^{\prime}} \\ {y^{\prime}} \\ {z^{\prime}} \\ 1 \\ \end{array} } \right] = f*\left[ \begin{gathered} x \hfill \\ y \hfill \\ z \hfill \\ 1 \hfill \\ \end{gathered} \right]$$

For a vertex translated with a vector *T*(*t*_0_, *t*_1_, *t*_2_), its translation matrix *f*_*t*_ can be shown as follows:

5 $$f_{t} = \left[ {\begin{array}{*{20}c} 1 & 0 & 0 & {t_{0} } \\ 0 & 1 & 0 & {t_{1} } \\ 0 & 0 & 1 & {t_{2} } \\ 0 & 0 & 0 & 1 \\ \end{array} } \right]$$

Then, the vertices’ Cartesian coordinate values of the face mesh can be computed according to Eqs. (4) and (5) based on *T*_*s*_.

#### Rotation

After the translation, the face mesh is rotated with the rotation transformation matrix *f*_*s*1_ to make the consistency between *N*_*s*_ and *N*_*x*_. The rotation axis and angle can be, respectively denoted with *A*_0_ and *α*_0_ that are, respectively calculated by *A*_0_ = *N*_*s*_ × *N*_*x*_ and *α*_0_ = arccos (*N*_*s*_ ∙ *N*_*x*_).

A rotation transformation matrix *f*_*r*_ can be computed as follows with the rotation axis and angle, which are, respectively denoted with *V*_*ax*_(*x*_*ax*_, *y*_*ax*_, *z*_*ax*_) and *α*^32^:

6 $$f_{r} = \left[ {\begin{array}{*{20}c} {xx + \cos \alpha } & {xy - zs} & {xz + ys} & 0 \\ {xy + zs} & {yy + cos\alpha } & {yz - xs} & 0 \\ {xz - ys} & {yz + xs} & {zz + \cos \alpha } & 0 \\ 0 & 0 & 0 & 1 \\ \end{array} } \right]$$

where $$r = \sqrt {x_{ax}^{2} + y_{ax}^{2} + z_{ax}^{2} }$$; $$xx = \left( {1 - \cos \alpha } \right) \cdot \frac{{x_{ax} }}{r} \cdot \frac{{x_{ax} }}{r}$$; $$xy = \left( {1 - \cos \alpha } \right) \cdot \frac{{x_{ax} }}{r} \cdot \frac{{y_{ax} }}{r}$$; $$xz = \left( {1 - \cos \alpha } \right) \cdot \frac{{x_{ax} }}{r} \cdot \frac{{z_{ax} }}{r}$$; $$yy = \left( {1 - cos\alpha } \right) \cdot \frac{{y_{ax} }}{r} \cdot \frac{{y_{ax} }}{r}$$; $$yz = \left( {1 - cos\alpha } \right) \cdot \frac{{y_{ax} }}{r} \cdot \frac{{z_{ax} }}{r}$$; $$zz = \left( {1 - \cos \alpha } \right) \cdot \frac{{z_{ax} }}{r} \cdot \frac{{z_{ax} }}{r}$$; $$xs = \frac{{x_{ax} }}{r} \cdot \sin \alpha$$; $$ys = \frac{{y_{ax} }}{r} \cdot \sin \alpha$$; $$zs = \frac{{z_{ax} }}{r} \cdot \sin \alpha$$.

Then, the vertices’ Cartesian coordinate values of the face mesh can be computed according to Eq. (4) based on *f*_*s*1_, which is calculated according to Eq. (6) based on *A*_0_ and *α*_0_.

After the translation and the rotation, the alignment result is shown in the right map of Fig. 7. According to the alignment, *N*_*s*_ is parallel to *N*_*x*_ of the myopic glasses.

### Coordinate system alignment

**Figure 8:**
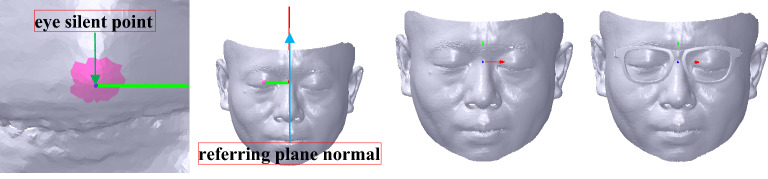
Coordinate system alignment. From left to right: silent point extraction of the eye with blue sphere, referring plane defined with red normal, coordinate system alignment result, coordinate system alignment result with showing the myopic glasses.

Coordinate system alignment contains *X* axis alignment, *XOZ* plane alignment and coordinate system translation. During *X* axis alignment, the face is translated to confirm its pupil distance point *V*_2_(*x*_2_, *y*_2_, *z*_2_) which is the silent point of the eye located in the pupil height line of the glasses frame. *V*_2_ can be extracted based on a descriptor named average weight curvature (AWC). The AWC of a vertex is calculated based on its curvature, coordinate value, and normal. Its computation can be shown as follows. First, the average coordinate value $\overline{V}(\overline{x},\overline{y},\overline{z})$ and the sum of the vertex normal *N*(*n*_0_, *n*_1_, *n*_2_) can be calculated according to the selected mesh. Second, each vertex’s coordinate deviation value *V*_*d*_(*x*_*d*_, *y*_*d*_, *z*_*d*_) can be obtained with its coordinate value subtracted by $\overline{V}$. Third, each vertex’s AWC can be denoted as *A*_*w*_, which is represented by *A*_*w*_ = *k*_1_^2^ + *k*_1_*k_2_ + *k*_2_^2^ + *V*_*d*_ ∙ *N*, where *k*_1_ and *k*_2_ are the maximum and minimum curvatures, respectively. Finally, the maximum value of the vertex’s *A*_*w*_ can be found and selected as *V*_2_, as shown with a blue sphere in the selected mesh marked with a pink mesh in the left map of Fig. 8. Then, the translated vector *T*_*c*0_ can be obtained with *T*_*c*0_(0, − *y*_2_, − *z*_2_). Therefore, the vertices’ Cartesian coordinate values of the face mesh can be computed according to Eqs. (4) and (5) based on *T*_*c*0_.

After *X* axis alignment, a referring plane *P*_*R*_ is created to rotate the face mesh to align to *XOZ* plane, whose normal can be represented with *N*_*y*_(0, 1, 0). *P*_*R*_ can be created by three vertices, namely, *V*_2_, *V*_2*a*_(*x*_2*a*_, *y*_2*a*_, *z*_2*a*_) and *V*_2*b*_(*x*_2*b*_, *y*_2*b*_, *z*_2*b*_). *V*_2*a*_ can be defined with *V*_2_ projected on *YOZ* plane by applying Eqs. (1), (2) and (3). *V*_2*b*_ can be presented with *V*_2*a*_ projected on the face mesh. Thus, *P*_*R*_ can be created with its plane coefficient denoted as *P*_*u*_[*p*_*u*0_ *p*_*u*1_ *p*_*u*2_ *p*_*u*3_]. Then, *P*_*R*_ normal *N*_*u*_(*p*_*u*0_, *p*_*u*1_, *p*_*u*2_) can be computed with *N*_*u*_ = (*V*_2_*V*_2*a*_ × *V*_2_*V*_2*b*_)/|| *V*_2_*V*_2*a*_ × *V*_2_*V*_2*b*_||_2_. *p*_*u*3_ can be obtained with *p*_*u*3_ = − *N*_*u*_ ∙ *V*_2_. In the end, *P*_*R*_ can be shown with its normal marked with a red line in the second-to-left map of Fig. 8. Then, the face mesh is rotated with transformation matrix *f*_*c*0_. The rotation axis and angle can be, respectively denoted with *A*_1_ and *α*_1_ that are, respectively calculated by Eq. (7) and *α*_1_ = arccos (*N*_*u*_ ∙ *N*_*y*_). Then, the vertices’ Cartesian coordinate values of the face mesh can be computed according to Eq. (4) based on *f*_*c*0_, which is calculated according to Eq. (6) based on *A*_1_ and *α*_1_.

7 $$A_{1} = \left\{ \begin{gathered} N_{x} ,(N_{u} \times N_{y} ) \cdot N_{x} > 0 \hfill \\ - N_{x} ,(N_{u} \times N_{y} ) \cdot N_{x} < 0 \hfill \\ \end{gathered} \right.$$

During the coordinate system translation, the distance between the face pupil distance point and the coordinate system of the glasses along *Z* axis is adjusted to keep a suitable distance between the glasses and the eye. It is usually that the distance set at 11 mm can make the try-on health and comfort come true. Thus, the translation vector *T*_*c*1_ can be denoted with *T*_*c*1_ (0, 0, − 11) to obtain an ideal score of M-DGE of GFS. Therefore, the vertices’ Cartesian coordinate values of the face mesh can be computed according to Eqs. (4) and (5) based on *T*_*c*1_. After the coordinate system alignment, the result is shown in the third-to-left and right maps of Fig. 8.

### Revised alignment

**Figure 9:**
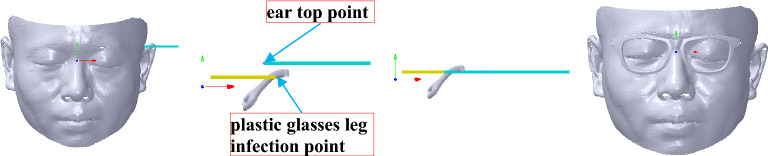
Revised alignment. From left to right: selection of the infection point of the plastic glasses leg and the top point of the ear with showing the face, the infection point of the plastic glasses leg and the top point of the ear with red and blue points and sublines through points along *X* axis, revised alignment result, revised alignment result with showing the face and the myopic glasses.

After the coordinate system alignment, the face has not been aligned to the myopic glasses at an accurate position, *e.g.*, the inconsistency between the inflection point of the plastic glasses leg (labelled with the red words in Fig. 2) and the top point of the ear that are marked with *V*_*L*_(*x*_*L*_, *y*_*L*_, *z*_*L*_) and *V*_*E*_(*x*_*E*_, *y*_*E*_, *z*_*E*_), respectively, as shown in Fig. 9 with the red and blue points. The ideal consistency is that *V*_*E*_ has the same coordinate value as *V*_*L*_ along *Y* axis.

**Figure 10:**
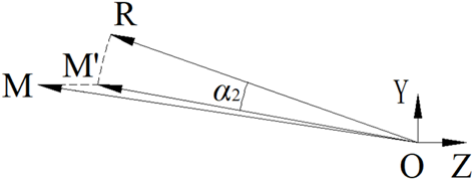
The rotation angle of the revised alignment.

Subsequently, the face mesh is rotated along a certain axis *A*_2_ to make *V*_*E*_ keep the same coordinate value as *V*_*L*_ along *Y* axis, as shown in Fig. 10.

In Fig. 10, points *R*(0, *y*_*E*_, *z*_*E*_) and *M*(0, *y*_*L*_, *z*_*L*_) are *V*_*E*_ and *V*_*L*_, respectively, which are projected on *YOZ* plane. After rotation with angle *α*_2_, *R* is rotated to *M*′(0, *y*_*E*_, *z*_*M*_′) to keep the same coordinate value of *Y* axis with *M*. As the length of *OR* is the same as that of *OM*′, that is, ‖*OR*‖_2_ = ‖*O M*′‖_2_, *M*′ can be computed on the same side of *M*. Thus, *A*_2_ can be obtained by *A*_2_ = (*OR* × *OM*′)/‖*OR* × *OM*′‖_2_, and *α*_2_ is calculated by *α*_2_ = arccos (*OR* ∙ *OM*′). Therefore, the face mesh can be computed according to Eq. (4) based on transformation matrix *f*_*R*_, which is calculated according to Eq. (6) based on *A*_2_ and *α*_2_. The result is shown in the third-to-left and right maps of Fig. 9.

## Glasses personalized design

### Glasses frame transformation

#### Frame transformation

The frame transformation can be summarized with two steps: translation and rotation.

##### Translation

**Figure 11:**
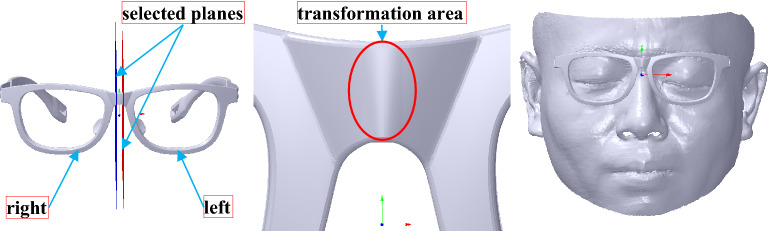
The frame translation. From left to right: the frame translation based on selected planes marked with blue and red planes, the frame translation result, the frame translation result showing the face.

The frame is translated to make the geometric central distance of the glasses satisfy the pupil distances of the left and right eyes. The translation is achieved by scaling the nose bridge within the selected planes that pass through vertices *V*_*CR*_(*x*_*CR*_, 0, 0) and *V*_*CL*_(*x*_*CL*_, 0, 0) and parallel to *YOZ* plane, as shown in the left map of Fig. 11, wherein *x*_*CR*_ and *x*_*CL*_ are less and more than zero, respectively. Furthermore, the scale of the nose bridge is only along *X* axis to maintain the frame feature. Suppose the geometric central points of the glasses and the pupil distance points of the face are, respectively marked with *V*_*GR*_(*x*_*GR*_, 0, 0), *V*_*GL*_(*x*_*GL*_, 0, 0), *V*_*ER*_(*x*_*ER*_, 0, 0) and *V*_*EL*_(*x*_*EL*_, 0, 0), the frame is translated with the translation matrix *f*_1_ and *f*_2_ out of the selected planes, and deformed with the scale matrix *f*_3_ and *f*_4_ within the selected planes. *V*_*GR*_ and *V*_*GL*_ are the right and left geometric central points of the glasses, respectively. *V*_*ER*_ and *V*_*EL*_ are the right and left pupil distance points of the face, respectively. According to the size of the glasses and the face mentioned before, *x*_*GR*_, *x*_*GL*_, *x*_*ER*_ and *x*_*EL*_ are − 33.55, 33.55, − 33 and 33, respectively. Thus, *f*_1_ and *f*_2_ can be computed according to Eq. (4) based on the vector *T*_*g*0_(− (*x*_*ER*_ − *x*_*GR*_), 0, 0) and *T*_*g*1_(*x*_*EL*_ − *x*_*GL*_, 0, 0). The scale transformation matrix *f*_*s*_ can be calculated by the scale vector *S*(*s*_*x*_, *s*_*y*_, *s*_*z*_) as follows:

8 $$f_{s} = \left[ {\begin{array}{*{20}c} {s_{x} } & 0 & 0 & 0 \\ 0 & {s_{y} } & 0 & 0 \\ 0 & 0 & {s_{z} } & 0 \\ 0 & 0 & 0 & 1 \\ \end{array} } \right]$$

As the deformation within the selected planes is only along *X* axis, *f*_3_ and *f*_4_ can be obtained by the scale vector *S*_0_((*x*_*ER*_ − *x*_*GR*_ +|*x*_*CR*_|)/|*x*_*CR*_|, 1, 1) and *S*_1_((*x*_*EL*_ − *x*_*GL*_ + *x*_*CL*_)/*x*_*CL*_, 1, 1) according to Eq. (8). Therefore, the transformation matrix *f*_0_ can be defined as follows:

9 $$f_{0} = \left\{ \begin{gathered} f_{1} ,x < x_{CR} \hfill \\ f_{3} ,x_{CR} \le x < 0 \hfill \\ f_{4} ,0 \le x \le x_{CL} \hfill \\ f_{2} ,x > x_{CL} \hfill \\ \end{gathered} \right.$$

Then, the frame mesh can be deformed according to Eq. (4) based on *f*_0_ to obtain an ideal score of M-GP of GFS, as shown in the middle and right maps of Fig. 11.

##### Rotation

**Figure 12:**
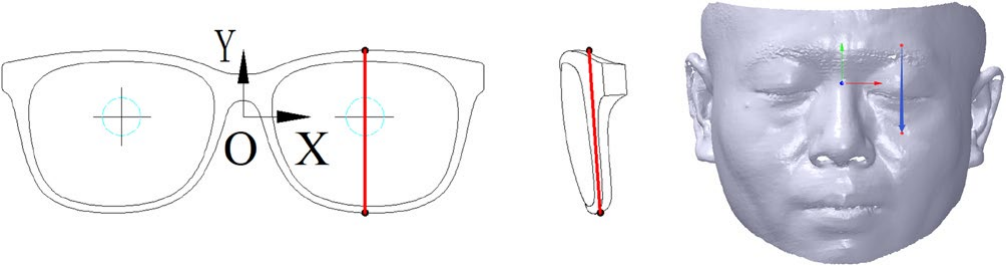
The dip line of the frame and the face. From left to right: frame, face.

The frame is rotated to maintain the consistency between the dip angles of the frame and the face, as shown in Fig. 12.

On the one hand, the dip line of the frame *L*_0_ is defined in two steps. First, it is defined by the plane passing through the geometric central point and paralleling to *YOZ* plane, as shown in the left map of Fig. 12. Second, it is made up of two points that are the inner vertex of the up and down frames in the section (Fig. 2), as shown in the left map of Fig. 12. On the other hand, the dip line of the face *L*_1_ can be shown in the right map of Fig. 12, wherein it is in the section with also passing through the geometric central point and paralleling to *YOZ* plane and made up of two points that are, respectively the vertex in the positon of the eyebrow and the cheek. If *L*_1_ and *L*_0_ are inconsistent, that is, *L*_1_ and *L*_0_ projected on *YOZ* plane are not parallel to each other, it may lead to the try-on discomfort, for instance, the contact between the glasses and the face with a great expression. Therefore, the frame is rotated along *X* axis with paralleling *L*_0_ to *L*_1_.

For the frame, *L*_0_ can be obtained by the section defined by the frame and the plane *P*_*GD*_ with coefficient *P*_1_[1 0 0 − *x*_*EL*_] that passes through *V*_*EL*_ and parallels *YOZ* plane. It can be divided into two steps: the intersection mesh selection and the intersection point computation. During the first step, the marked mesh *M*_*m*_ is selected when its *X* coordinate value of the vertex is more than *x*_*EL*_. Then, the intersection mesh *M*_*IG*_ is selected with 1-ring neighborhood adjacent triangles of the boundary vertices of *M*_*m*_. Thus, the intersection edges can be computed with their different vertices’ sign distances on *P*_*GD*_.

**Figure 13:**
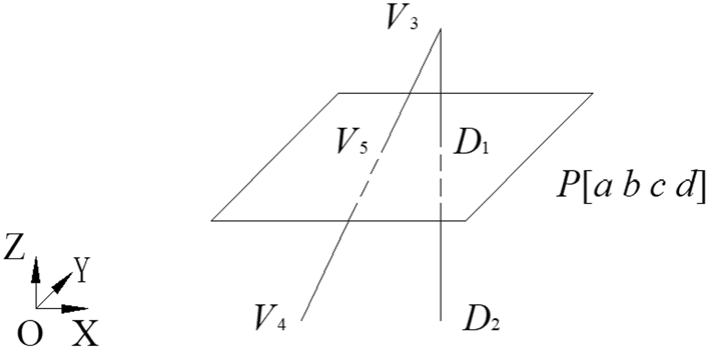
The intersection point between line and plane.

Suppose a line and a plane have coefficient *P*[*a b c d*]. The intersection point can be computed as shown in Fig. 13, wherein the intersection line consists of two vertices *V*_3_(*x*_3_, *y*_3_, *z*_3_) and *V*_4_(*x*_4_, *y*_4_, *z*_4_), and their projection points are marked with *D*_1_(*x*_*d*1_, *y*_*d*1_, *z*_*d*1_) and *D*_2_(*x*_*d*2_, *y*_*d*2_, *z*_*d*2_), respectively. Obviously, the scale at the projection line divided by *D*_1_ is the same as the intersection line divided by the intersection point *V*_*5*_(*x*_*5*_, *y*_*5*_, *z*_*5*_), as shown in Eq. (10).

10 $$\frac{{V_{3} V_{5} }}{{V_{3} V_{4} }} = \frac{{V_{3} D_{1} }}{{V_{3} D_{2} }} = \frac{{\left\| {V_{3} D_{1} } \right\|_{2} }}{{\left\| {V_{3} D_{2} } \right\|_{2} }} = \frac{{ax_{3} + by_{3} + cz_{3} + d}}{{a(x_{3} - x_{4} ) + b(y_{3} - y_{4} ) + c(z_{3} - z_{4} )}} = \lambda$$

where *λ* is the scale factor. Then, *V*_*5*_ can be compute as follows:

11 $$OV_{5} = OV_{3} + V_{3} V_{5} = OV_{3} + \lambda *V_{3} V_{4}$$

**Figure 14:**
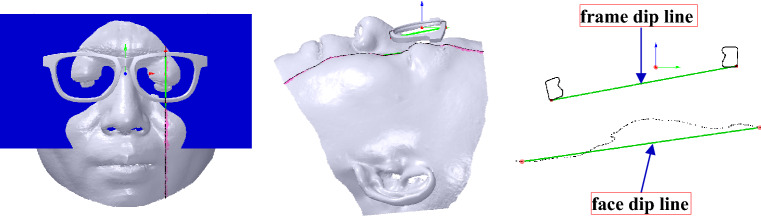
The dip line of the glasses and the face are marked with green lines. From left to right: the front view, the side view, the side view without the fame and face.

Therefore, the intersection points can be computed based on the intersection edges and *P*_1_, as shown with the black vertices in Fig. 14, wherein the pink mesh in the glasses is *M*_*IG*_. Subsequently, *L*_0_ is created with its vertices selected manually in the intersection points, as shown with the red vertices and green line in Fig. 14, wherein the vertices in the up and down frames are respectively marked with *V*_10_(*x*_10_, *y*_10_, *z*_10_) and *V*_11_ (*x*_11_, *y*_11_, *z*_11_).

Similar to *L*_0_, the intersection mesh *M*_*IF*_ and points *P*_*IF*_ can be obtained, as shown in Fig. 14, wherein the pink mesh in the face is *M*_*IF*_ and the black vertices are *P*_*IF*_. *L*_1_’s vertices are manually and initially selected in the position of the eyebrow and the cheek, as shown with the red vertices and green line in Fig. 14, wherein the vertices in the position of the eyebrow and the cheek are marked with *V*_20_(*x*_20_, *y*_20_, *z*_20_) and *V*_21_(*x*_21_, *y*_21_, *z*_21_), respectively. It is initial, as the vertices selected manually are inaccurate and should be adjusted in the next step. The adjustment is conducted to find the ideal vertices by rotating the plane through *L*_1_ along *N*_*x*_, as shown with the blue plane in Fig. 14, wherein the plane passes through *V*_20_, *V*_21_ and *V*_22_ (0, *y*_21_, *z*_21_).

**Figure 15:**
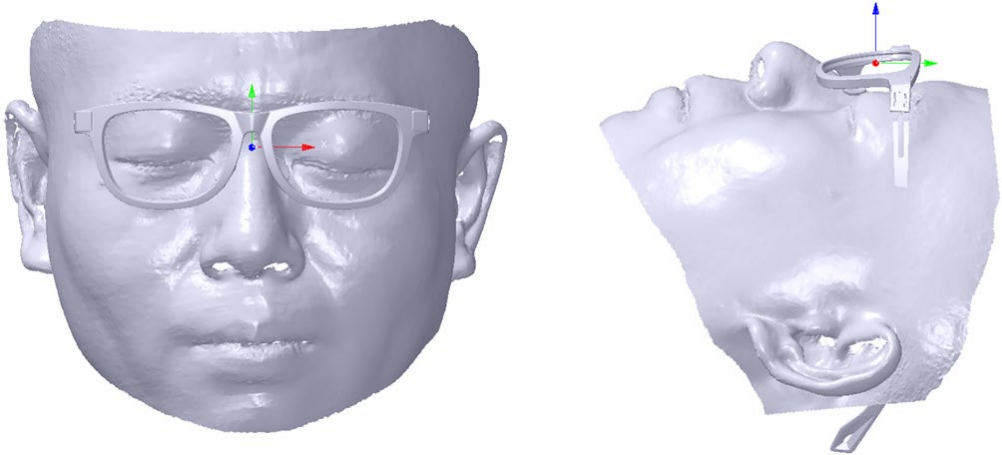
The frame rotation result. From left to right: the front view, the side view.

After obtaining *L*_0_ and *L*_1_, *L*_0_ can be rotated to parallel to *L*_1_ with obtaining an ideal M-DGF score of GFS. Then, the vertices can be revised with *V*_10_(0, *y*_10_, *z*_10_), *V*_11_(0, *y*_11_, *z*_11_), *V*_20_ (0, *y*_20_, *z*_20_) and *V*_21_ (0, *y*_21_, *z*_21_). The rotation angle *α*_3_ can be computed by *α*_3_ = arccos (*V*_10_*V*_11_ ∙ *V*_20_*V*_21_). Subsequently, the vertices’ Cartesian coordinate values of the frame mesh can be computed according to Eq. (4) based on the rotation transformation matrix *f*_5_, which is calculated according to Eq. (6) based on *N*_*x*_ and *α*_3_, as shown in Fig. 15.

#### Nose pad transformation

Nose pad transformation can be summarized with two procedures: translation and rotation.

##### Translation

**Figure 16:**
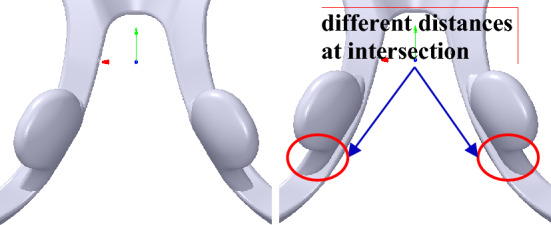
The translation of the nose pad along *Y* direction. From left to right: origin nose pad, translation.

The nose pad can be translated along *X*, *Y* and *Z* axis with the transformation matrix *f*_*n0*_ based on the translation vector *T*_*n*0_(*x*_*n*0_, *y*_*n*0_, *z*_*n*0_). However, if the nose pad is translated along *Y* axis, there may be a connection relationship problem between the nose pad and the frame, as shown in Fig. 16, wherein the different distances at the intersection may affect the appearance of the glasses.

**Figure 17:**
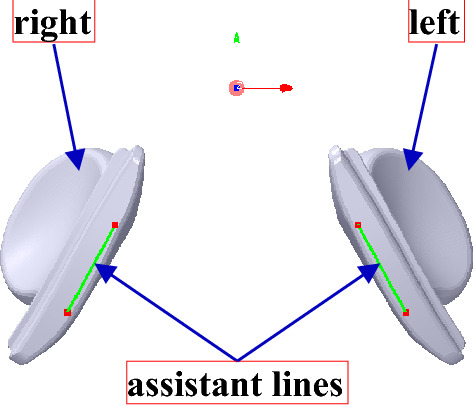
The assistant lines of the nose pad.

**Figure 18:**
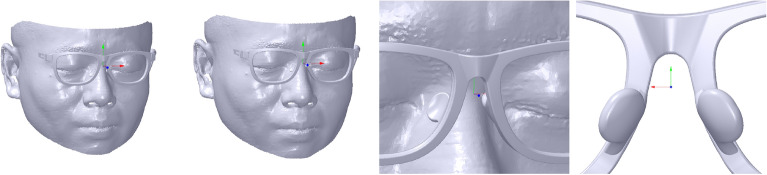
The translation of the nose pad. From left to right: original nose pad, the translation of the nose pad, the translation of the nose pad with a partially enlarged view, and the translation of the nose pad with a partially enlarged view without showing the face.

Two assistant lines are set in the nose pad to solve the problem, as shown in Fig. 17, wherein the lines are made up of two protruding points marked with *V*_30_(*x*_30_, *y*_30_, *z*_30_) and *V*_31_(*x*_31_, *y*_31_, *z*_31_), *V*_40_(*x*_40_, *y*_40_, *z*_40_) and *V*_41_(*x*_41_, *y*_41_, *z*_41_) that are in the left and right nose pads, respectively. Then, *T*_*n*0_ can be defined along *X* axis, the assistant lines and *Z* axis by Eq. (12).

12 $$T_{n0} = \left\{ \begin{gathered} (x_{n0} ,0,0),x > 0 \hfill \\ ( - x_{n0} ,0,0),x < 0 \hfill \\ (x_{a} ,y_{a} ,z_{a} ),x > 0 \hfill \\ (x_{b} ,y_{b} ,z_{b} ),x < 0 \hfill \\ (0,0,z_{n0} ) \hfill \\ \end{gathered} \right.$$

where *T*_*a*_(*x*_*a*_, *y*_*a*_, *z*_*a*_) and *T*_*b*_(*x*_*b*_, *y*_*b*_, *z*_*b*_), the translation vectors of the left and right nose pads along the left and right assistant lines, respectively, can be calculated as follows:

13 $$x_{a} = \delta \cdot \cos (\arccos (V_{30} V_{31} \cdot N_{x} ))$$

14 $$y_{a} = \delta \cdot \cos (\arccos (V_{30} V_{31} \cdot N_{y} ))$$

15 $$z_{a} = \delta \cdot \cos (\arccos (V_{30} V_{31} \cdot N_{z} ))$$

16 $$x_{b} = \delta \cdot \cos (\arccos (V_{40} V_{41} \cdot N_{x} ))$$

17 $$y_{b} = \delta \cdot \cos (\arccos (V_{40} V_{41} \cdot N_{y} ))$$

18 $$z_{b} = \delta \cdot \cos (\arccos (V_{40} V_{41} \cdot N_{z} ))$$

where *δ* is the translation distance and *N*_*z*_ is *Z* axis vector denoted as *N*_*z*_(0, 0, 1). Then, the vertices’ Cartesian coordinate values of the nose pad mesh can be computed according to Eqs. (4) and (5) based on *T*_*n*0_, as shown in Fig. 18.

##### Rotation

The nose pad is rotated to match the nose along the assistant lines. The transformation matrices *f*_*n*1_ and *f*_*n*2_ are for the left and right nose pads, respectively. Their rotation axes can be defined with *A*_3_ and *A*_4_, which can be denoted as *A*_3_ = *V*_30_*V*_31_ and *A*_4_ = *V*_40_*V*_41_, respectively.

**Figure 19:**
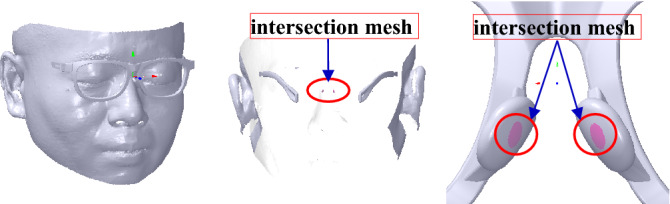
The rotation of the nose pad. From left to right: the front view, a partial enlarged and back view, a partial enlarged and back view without showing the face.

Then, the vertices’ Cartesian coordinate values of the nose pad mesh can be computed according to Eq. (4) based on *f*_*n*1_ and *f*_*n*2_, which is calculated according to Eq. (6) based on *A*_3_ and *A*_4_ with an assigned angle value, as shown in Fig. 19.

**Figure 20:**
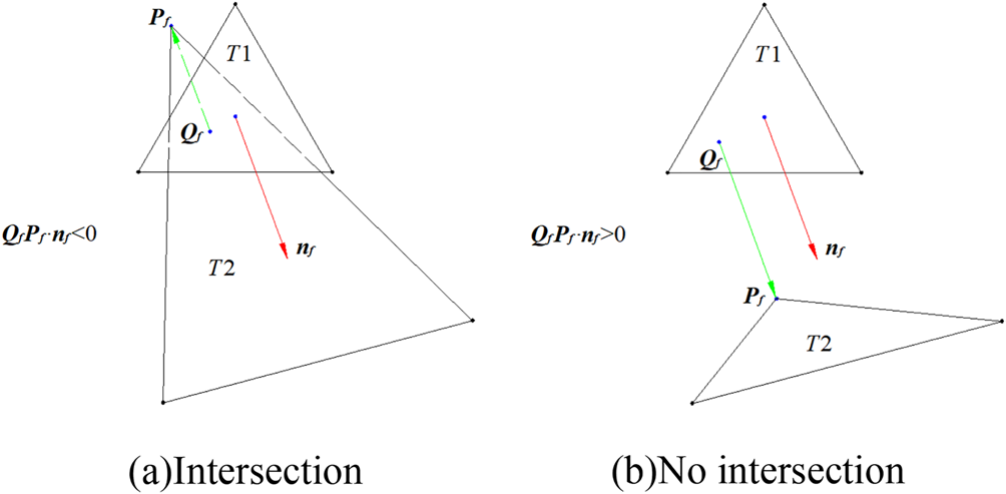
The statement between two triangles.

Furthermore, it is necessary to stop the rotation when there is much more intersection mesh between the nose pad and the nose to obtain an ideal M-NN score of GFS. The intersection mesh can be extracted as shown in Fig. 20, wherein there are two triangles T1 and T2. The vertex *Q*_*f*_ is the vertex *P*_*f*_ of T2 projected on T1 with normal *n*_*f*_. Obviously, if *Q*_*f*_*P*_*f*_ ∙ *n*_*f*_ < 0, the triangles may intersect each other. Therefore, after every rotation, the approach proposed above is applied to computing the intersection mesh between the nose pad and the nose, as shown in Fig. 19, wherein the pink mesh is the intersection mesh.

#### Pile head transformation

**Figure 21:**
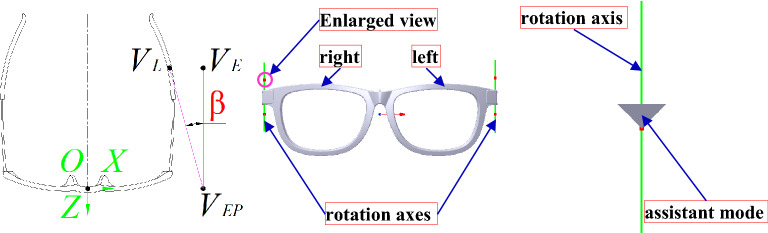
The transformation definition of the pile head. From left to right: the definition of angle between the leg and the face, the rotation axes of the pile head marked with green lines, the rotation axis of the pile head with a partial enlarged view.

Because the distance between the left and right legs affects the myopic glasses try-on comfort, it is important to have a suitable distance. The shorter distance may affect try-on comfort with tightness. The longer distance may lead to the fall of the glasses. In this paper, the distance is adjusted to match the face by rotating the pile head with a definition angle *β*. *β* is the angle between the leg and face, as shown in the left map of Fig. 21, which is defined to evaluate the try-on comfort between the glasses and the face. According to the definition, *β* is made up of three points, which are *V*_*E*_, *V*_*L*_ and *V*_*EP*_(*x*_*E*_, *y*_*E*_, 0) with *V*_*E*_ projected on *XOY* plane. Hence, *β* can be expressed with *β* = arccos (*V*_*EP*_*V*_*E*_ ∙ *V*_*EP*_*V*_*L*_).

To ensure that the consistency between *V*_*E*_ and *V*_*L*_ along *Y* direction cannot be changed, the pile head is only limited to rotate along the vertical direction. The rotation axis is approximately the axis of the round corner at the pile head, as shown with green lines in the middle map of Fig. 21, wherein the axes are made up of the vertices marked with *V*_50_(*x*_50_, *y*_50_, *z*_50_) and *V*_51_(*x*_51_, *y*_51_, *z*_51_), *V*_60_ (*x*_60_, *y*_60_, *z*_60_) and *V*_61_(*x*_61_, *y*_61_, *z*_61_) that are in the right and left assistant modes of the pile head, respectively. For instance, the assistant mode is a cone located in the right pile head, as shown in the right map of Fig. 21, wherein the red point is *V*_50_. The transformation matrices *f*_*p*0_ and *f*_*p*1_ are for the right and left pile heads, respectively. Their rotation axes can be defined with *A*_5_ and *A*_6_, which can be denoted as *A*_5_ = *V*_50_*V*_51_ and *A*_6_ = *V*_60_*V*_61_, respectively. Then, the vertices’ Cartesian coordinate values of the pile head mesh and metal and plastic glass leg mesh can be computed according to Eq. (4) based on *f*_*p*0_ and *f*_*p*1_, which is calculated according to Eq. (6) based on *A*_5_ and *A*_6_ with an assigned angle value.

**Figure 22:**
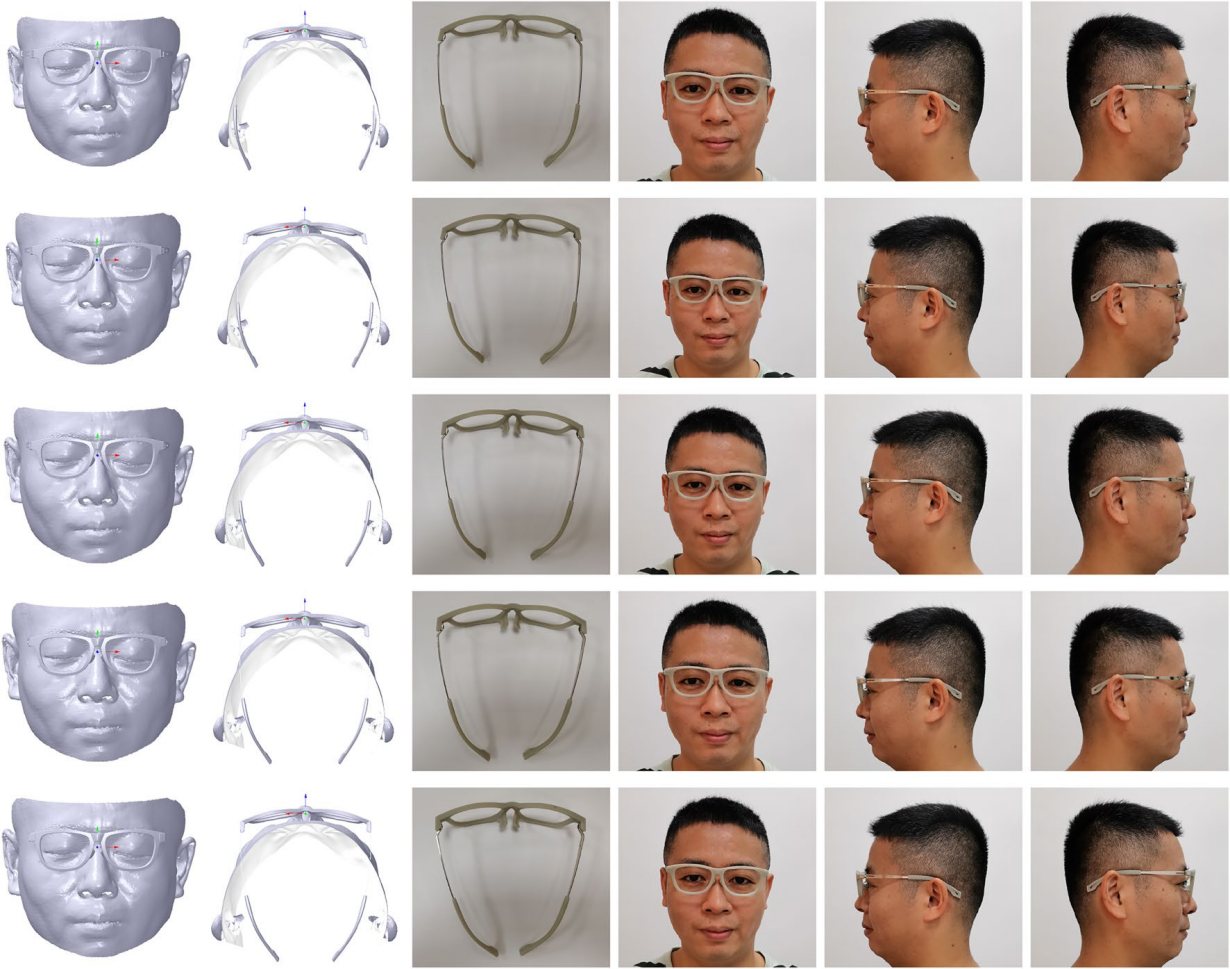
Try-on results of pile head rotation. From left to right: the front view, the vertical view, the printed glasses, the front view, the left view, the right view. From top to bottom: *β* with 3°, 6°, 9°, 12° and 15°.

To obtain an ideal *β*, a series of pile head transformation experiments are conducted with *β* values of 3°, 6°, 9°, 12° and 15°, as shown in Fig. 22. Try-on results show that when *β* is more than 9°, as the distance between the left and right leg is so short that the try-on comfort is bad with serious tightness. When *β* is between 3° and 6°, the try-on result is good with the good try-on comfort of suitable tightness for obtaining an ideal M-PF score of GFS. In this paper, *β* with 6° is selected for conducting the application and comparison.

### Plastic glasses leg option

**Figure 23:**
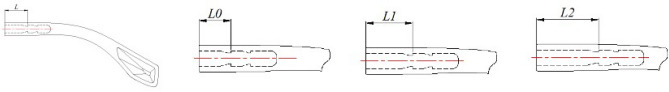
A series of glasses leg. From left to right: glasses leg with *L* variable match depth, glasses leg with *L*0 match depth, glasses leg with *L*1 match depth, glasses leg with *L*2 match depth.

The plastic glass legs’ match with the ear may affect try-on comfort and tightness. The glasses leg is designed with three series of difference matches with the metal glasses leg to adjust the match with the face, as shown with variable match depth *L* in Fig. 23, wherein the match depths between the plastic and the metal glasses leg are different, which are *L*0, *L*1 and *L*2, respectively. A certain series of plastic glasses legs can be selected to keep the match of length between the myopic glasses and the face, that is, the consistency of *V*_*E*_ and *V*_*L*_ along *Z* axis. In this paper, *L* is selected to be 13.5 mm, and the distance between *V*_*E*_ and *V*_*L*_ along *Z* axis is 3.06 mm, which can obtain an ideal score of the M-LGF of GFS.

### Glasses personalized mark

**Figure 24:**
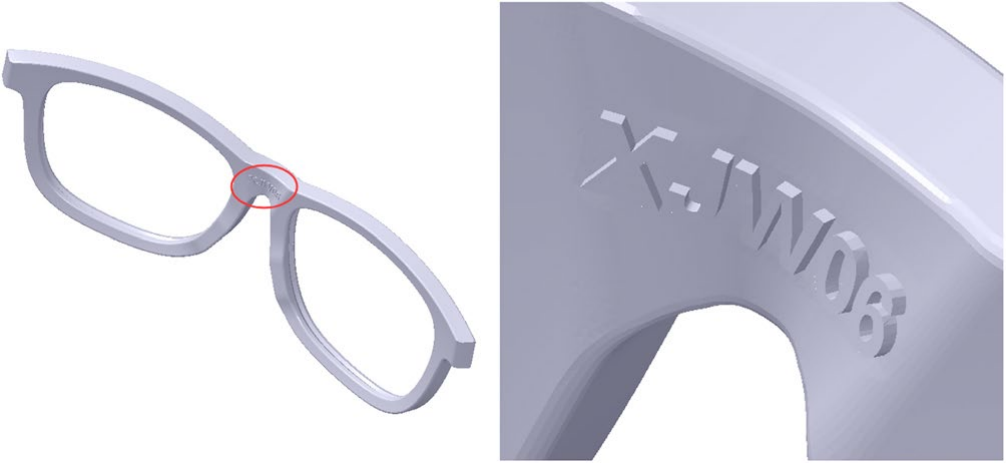
The mark in the frame. From left to right: the frame, the frame with an enlarged view.

**Figure 25:**
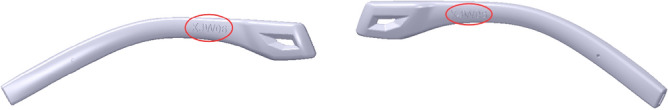
The mark in the plastic glasses legs. From left to right: the right leg, the left leg.

In the course of post processing, it is difficult to identify the parts belonging to the same pair of the myopic glasses, especially for the same style of the glasses. Therefore, a list of number features are marked on the frame and the plastic glasses legs by applying the feature reuse approach^33^, as shown in Fig. 24 and Fig. 25.

## Glasses manufacturing

**Figure 26:**
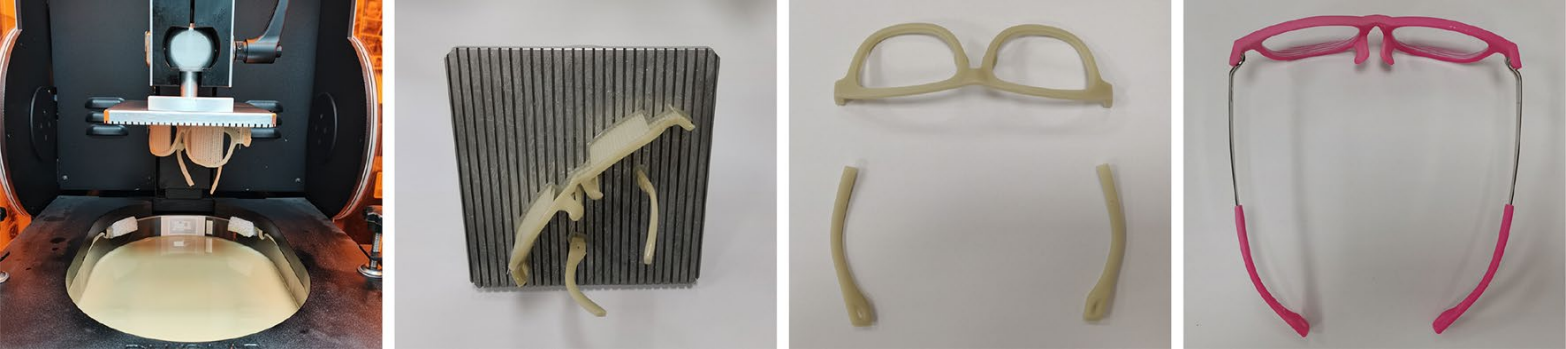
Glasses manufacturing. From left to right: the glasses fabricated by the 3D printer, the glasses with supports in the 3D printer platform, the glasses supports cleared manually, and the glasses polished, painted and assembled.

After glasses alignment and personalized design, the designed myopic glasses were fabricated with DM500 material by a 3D printer in XFAB 3500SD mode from Italy, as shown in Fig. 26. Then, the glasses were cleaned by an ultrasonic cleaner, and supports were cleared manually. Subsequently, the glasses were polished by a grinding mill and painted with a selected colour. Finally, the lens is processed by edge grinding machine according to the size of glasses frame, and assembled to the glasses frame manually.

**Figure 27:**
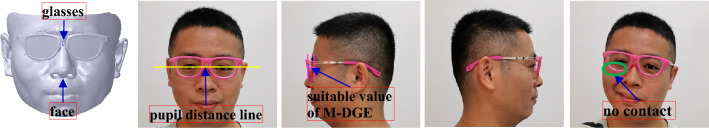
Try-on results of the myopic glasses based on our method. From left to right: the scanned face with glasses, the front view, the left view, the right view, the front view with a great expression.

**Figure 28:**
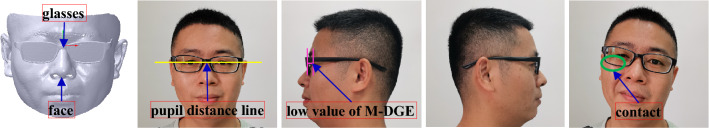
Try-on results of the myopic glasses based on the commercial glasses. From left to right: the scanned face with glasses, the front view, the left view, the right view, the front view with a great expression.

The try-on results of the myopic glasses and GFS are shown in Fig. 27 and Table 3, respectively. The GFS is computed by the method mentioned above based on the scanned face with the glasses, which is scanned first. Then, the whole glasses are aligned to the glasses worn on the face. As the glasses are adjusted based on the individual faces, M-DGE, M-GP, M-NN, M-PF, M-DGF and M-LGF can be approximately satisfied, although there might be errors compared to the ideal values because of manufacturing and assembly errors. A perfect GFS can be achieved to ensure the health and comfort of try-ons. Specifically, the down frame of the glasses cannot contact the face with a great expression shown in the right map of Fig. 27, compared to the commercial glasses shown in the right map of Fig. 28, where the down frame of the glasses can contact the face with a great expression. The reason is that the commercial glasses are based on experience without concerning the individual face, which can cause the imperfect GFS, as shown in Table 3. Therefore, the commercial glasses can usually fall from the nose to change the match between the pupil distance line and the geometric central line with the improper M-NN, and the glasses can contact the face with a great expression due to the great error of M-DGE and the bad M-DGF, leading to an uncomfortable try-on result.

**Table 3:** GFS of our method and the commercial glasses.

Methods	M-DGE	M-GP	M-NN	M-PF	M-DGF	M-LGF	AG
**Our method**							
Value	11.12	66	–	8.91	2.72	3.06	–
Error	0.12	0	–	2.91	2.72	0	–
**The commercial glasses**							
Value	6.01	–	–	7.15	10.43	4.44	–
Error	4.99	–	–	1.15	10.43	0	–

## Results and discussion

**Figure 29:**
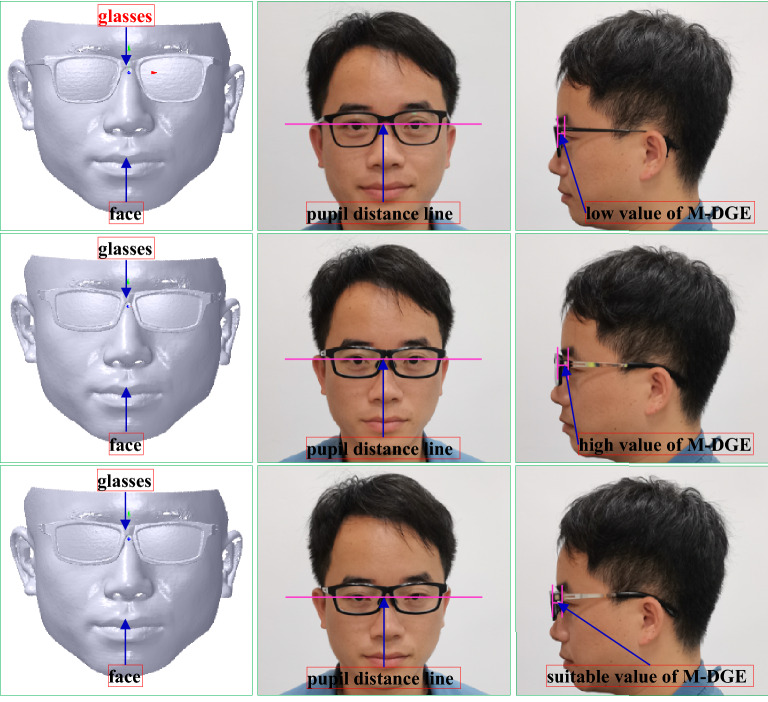
The try-on results of the myopic glasses of example 1. From left to right: the scanned face with glasses, the front view, the left view. From top to bottom: commercial glasses, the method of Liu^10^, our method.

**Figure 30:**
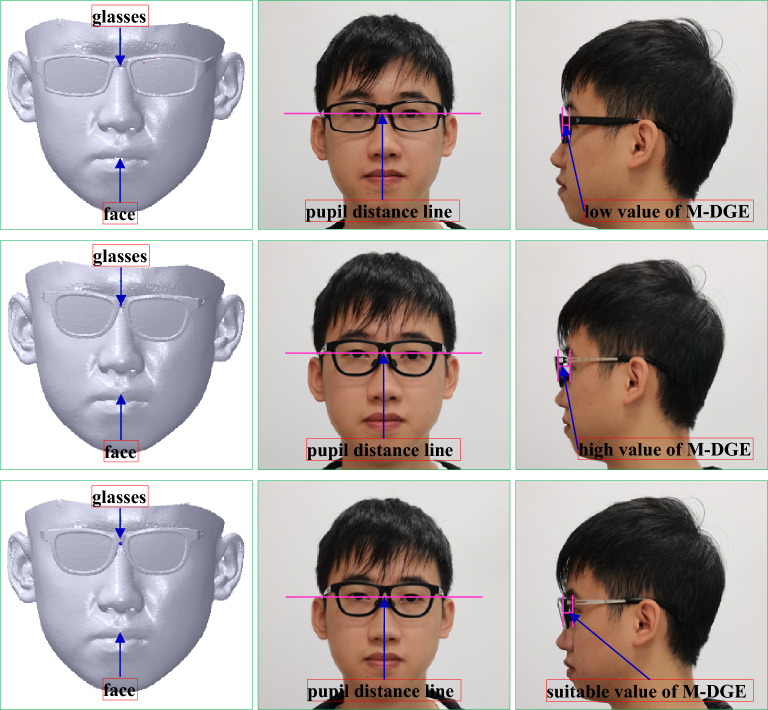
The try-on results of the myopic glasses of example 2. From left to right: the scanned face with glasses, the front view, the left view. From top to bottom: commercial glasses, the method of Liu^10^, our method.

**Figure 31:**
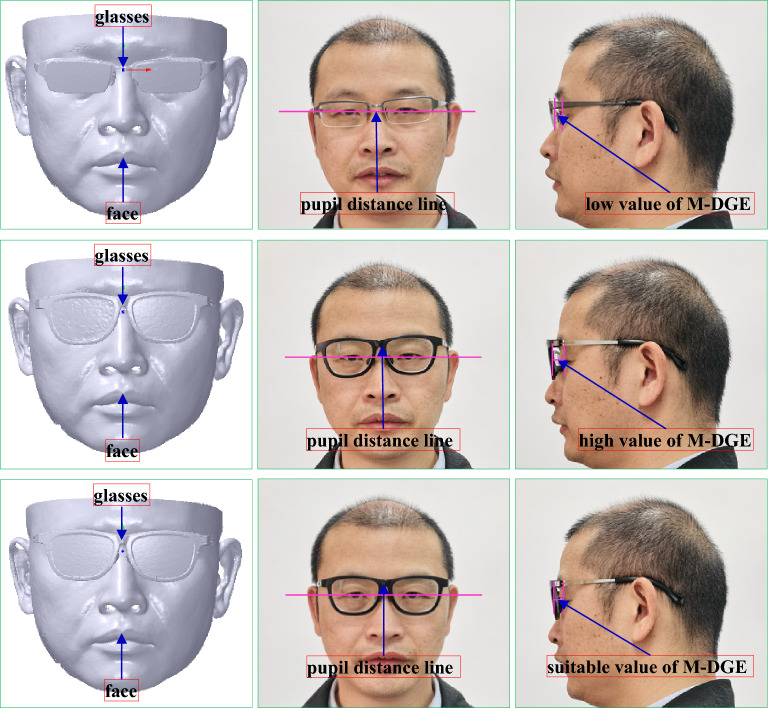
The try-on results of the myopic glasses of example 3. From left to right: the scanned face with glasses, the front view, the left view. From top to bottom: commercial glasses, the method of Liu^10^, our method.

To test the effect and robustness of our algorithm, we carry out our method to design the myopic glasses for comparison with the commercial glasses with forward design and the Liu^10^ method. The feature sizes of the example face and the selected myopic glasses are shown in Table 4, and the try-on results are shown in Figs. 29, 30 and 31 and Tables 5, 6 and 7. The method of GFS computation is mentioned in “Glasses manufacturing”.

**Table 4:** Feature sizes of the example face and selected myopic glasses.

	Face					Glasses	
	Myopic degree		Pupil distance		Width	Geometric central distance	Width
	Left	Right	Left	Right			
Example 1	250	175	34	33	139	70	141
Example 2	200	150	29	29	130	63	128
Example 3	500	550	33	33	150	73	147

**Table 5:** GFS of example 1 with the commercial glasses, the Liu^10^ method, and our method.

Item	Commercial glasses			The method of Liu^10^			Our method		
	Value	Error	Score	Value	Error	Score	Value	Error	Score
M-DGE	7.46	3.54	11	12.77	1.77	18	10.63	0.37	23
M-GP	–	–	15	67	0	25	67	0	25
M-NN	–	–	6	–	–	9	–	–	15
M-PF	2.07	0.93	9	4.34	0	10	6.97	0.97	9
M-DGF	4.87	4.87	7	5.45	5.45	7	1.44	1.44	9
M-LGF	1.90	0	10	0.49	0	10	3.86	0	10
AG	–	–	5	–	–	2	–	–	1
Total score	Total		63	Total		81	Total		92

**Table 6:** GFS of example 2 with the commercial glasses, the Liu^10^ method, and our method.

Item	Commercial glasses			The method of Liu^10^			Our method		
	Value	Error	Score	Value	Error	Score	Value	Error	Score
M-DGE	7.28	3.72	10	12.98	1.98	17	11.80	0.80	22
M-GP	–	–	15	60	2	21	60	2	21
M-NN	–	–	6	–	–	9	–	–	15
M-PF	6.72	0.72	9	8.26	2.26	8	8.72	2.72	7
M-DGF	2.47	2.47	9	5.94	5.94	7	2.99	2.99	8
M-LGF	1.57	0	10	2.83	0	10	4.13	0	10
AG	–	–	5	–	–	2	–		1
Total score	Total		64	Total		74	Total		84

**Table 7:** GFS of example 3 with the commercial glasses, the Liu^10^ method, and our method.

Item	Commercial glasses			The method of Liu^10^			Our method		
	Value	Error	Score	Value	Error	Score	Value	Error	Score
M-DGE	8.59	2.41	15	13.59	2.59	15	10.62	0.38	23
M-GP	–	–	15	70	4	17	70	4	17
M-NN	–	–	6	–	–	9	–	–	15
M-PF	3.13	0.13	9	5.91	0	10	4.55	0	10
M-DGF	4.85	4.85	7	2.94	2.94	8	1.92	0	9
M-LGF	1.84	0	10	0.86	0	10	4.24	0	10
AG	–	–	5	–	–	2	–		1
Total score	Total		67	Total		71	Total		85

Example 1 has different pupil distances. For the commercial glasses, the myopic glasses are assembled after optometry and measuring the pupil distance of the face. Obviously, AG can be perfect. M-GP can probably be satisfied, as the glasses are selected for fitting the individual face approximately. M-LGF can have a better match with the face. However, the glasses are designed and manufactured based on experience without concerning the individual face, and M-DGE, M-NN, M-PF and M-DGF cannot be well designed. The result is that the glasses can usually fall from the nose to change the match between the geometric central line and the pupil distance line with improper M-NN, M-PF and bad M-DGF, which can lead to the discomfort of the try-on results. These factors can cause great errors and a low GFS score of 63, leading to a poor MGIF.

For the Liu^10^ method, the pupil distance of the face, width and height of the nose, and width and length of the face were measured to adjust the myopic glasses. M-GP, M-PF and M-LGF can be satisfied. However, there are some match problems in MGIF. M-DGE cannot be well designed. Although the nose pad cannot slide from the nose, M-NN cannot be adjusted well, because it is only based on size without considering the shape of the face and the try-on comfort. Moreover, the dip angle of glasses cannot be adjusted to match the face. As the frame and the pile head are adjusted to match the face, there may be a small relationship problem in the pile heads. With slightly low M-DGE and M-NN scores, the match between the nose pad of the glasses and the nose of the face cannot be good, which can lead to an imperfect try-on comfort. The overall score of GFS is 81.

With regard to our method, because the myopic glasses are first aligned to the individual face considering the distance between the glasses and the eye, M-DGE can be approximately satisfied. Then, the nose bridge is translated to confirm M-GP, and the frame is rotated according to the dip angle of the face. Subsequently, the nose pad is translated and rotated to the nose based on the shape of the nose, and the pile head is rotated to keep a proper angle between the plastic glasses leg and the ear. Finally, a suitable plastic glasses leg is selected to ensure that the length between the glasses and face matches. However, the frame and the pile head are translated and rotated to match the face, and the connection relationship at the frame and the plie head cannot be perfect. In a word, the whole score of GFS is up to 92. Compared to the commercial glasses and the Liu^13^ method, our method can fully consider the try-on health and comfort. Therefore, the highest GFS score can be obtained with the best MGIF, and a perfect try-on glasses result can be achieved.

Example 2 has a slightly smaller pupil distance compared to the adult size of 62–68 mm^23^ and a slightly smaller face width compared to the adult size of 137–149 mm^34^. The commercial glasses cannot perfectly make the need for this feature and match the individual face well with GFS 64, leading to a bad MGIF. For the Liu^10^ method, GFS is 74. For our method, although the glasses frame is translated by 3 mm with an error of M-GP 2 mm, it can satisfy the standard under the condition of the myopic degree^35^. The glasses can be adjusted to a small pupil distance and match the face features with GFS 84, which can obtain the best MGIF compared to the commercial glasses and the Liu^10^ method.

The face width of example 3 is slightly larger compared to the adult size of 137–149 mm^34^. For the commercial glasses, it is difficult to design the glasses to match the features with GFS 67, which can cause the try-on health and comfort problems. To the method of Liu^10^, GFS is 71. For our method, although the glasses frame is translated by 3 mm with an error of M-GP 4 mm, it can satisfy the standard under the condition of the myopic degree^35^. As our algorithm can design the glasses based on the size of the face, the width and other features of the glasses can match well to the face. The GFS is 85, and MGIF can be improved greatly to obtain the best and most comfortable try-on compared to the commercial glasses and the Liu^10^ method.

## Conclusion

In this paper, a novel digital design and evaluation for additive manufacturing of personalized myopic glasses approach is presented to design the myopic glasses based on the individual face according to a novel evaluation descriptor GFS. As the myopic glasses are adjusted to the individual face based on the face features, an ideal GFS score can be obtained, which can ensure that MGIF can be greatly improved to enhance the try-on health and comfort, especially for the faces with asymmetric features.

Although the proposed approach can make the myopic glasses personalized deign come true, it has limitations in efficiency and accuracy. For the efficiency, the approach is based on some interactive operations in the process of glasses alignment and glasses personalized deign, which can lead to slightly low efficiency. For the accuracy, some face or glasses features are selected manually for alignment or design. There is inevitably an error between the practical and the ideal features that may affect the accuracy of alignment or design and lead to an impact on MGIF.

Future work can turn to these problems to carry out glasses alignment and personalized deign by decreasing the interactive operation. As there are some similar and obvious features to the face and glasses, these features may be detected based on artificial neural networks. According to the detected features and training samples, glasses alignment and personalized deign can be carried out rapidly and effectively to improve the approach efficiency and accuracy.

## Data Availability

The datasets generated and analysed during the current study are not publicly available due to the personal privacy of the participants in the study, but are available from the corresponding author on reasonable request.
